# Need assessment of medical school curriculum for MOOCs: perspectives of instructors and students of Shiraz University of Medical Sciences

**DOI:** 10.1186/s12909-024-05102-0

**Published:** 2024-02-13

**Authors:** Zahra Farhadi, Eisa Rezaei, Leila Bazrafkan, Mitra Amini, Nahid Zarif Sanaiey, Reza Barati-Boldaji, Manoosh Mehrabi

**Affiliations:** 1 Medical Education, Shiraz University of Medical Sciences, Shiraz, Iran; 2 Department of Educational Technology in Medical Sciences, Smart University of Medical Sciences, Tehran, Iran; 3 Clinical Education Research Center, Shiraz University of Medical Sciences, Shiraz, Iran; 4 Department of E-Learning in Medical Sciences, Virtual School (Center of Excellence for E-Learning in Medical Sciences), Shiraz University of Medical Sciences, Third Floor, Sina & Sadra Hall, Neshat Street, Shiraz, Iran; 5 Public Health Nutrition, Gastroenterohepatology Research Center, Shiraz University of Medical Sciences, Shiraz, Iran

**Keywords:** Educational needs assessment, Delphi technique, Medical students

## Abstract

**Introduction:**

Designing, developing, and implementing a course without assessing and prioritizing instructional needs may result in inefficiency due to the disregard for the actual needs of the target population. The present study aimed to determine and prioritize medical students’ instructional needs regarding Massive Open Online Courses (MOOCs) at Shiraz University of Medical Sciences.

**Methods:**

This survey study was carried out in three stages (2020–2021) using the Delphi technique. Purposive and snowball sampling methods were used to select the instructors. The students were selected through simple random sampling. The first round of the Delphi technique involved a questionnaire consisting of one open-ended question, completed by 49 basic/clinical faculty members and 47 senior medical students. In the second round, a 5-point Likert scale-based questionnaire was used to prioritize the instructional needs. The reliability of the questionnaire was verified by Cronbach’s alpha coefficient. In the third round, a focus group was used. A total of six expert faculty members and one senior medical student were invited to the focus group session to prioritize the needs. Data were analyzed using Friedman’s non-parametric ranking test in SPSS version 26.

**Results:**

Ten instructional needs priorities were extracted, including common pharmacotherapies (antibiotics and narcotics), prescriptions, physiology, anatomy, physical examination, electrocardiography interpretation, radiography, computed tomography scans, serum electrolyte disorders, and cardiovascular and internal (endocrine and metabolic) diseases. The chi-squared calculated value (715.584) indicated a significant difference in the importance of the questionnaire’s questions (*P* < 0.001). These questions did not have equal value, and the importance, from the respondent’s point of view and the observed distribution of ranks, was not the output of a random factor.

**Conclusions:**

The findings of this study can be used to design MOOCs, revise instructional programs, and adapt the curriculum to meet the needs of general practitioners, which will, in turn, help meet the medical needs of the general population.

## Background

Electronic learning (e-learning) is integral to medical education, especially in developed countries [1]. In basic medical sciences, complementary education by e-learning can help improve the quality of learning and clinical skills [2] and the long-term retention of information by students [3]. However, the effectiveness of e-learning depends on several factors. One important factor is the integration of technology tools into the curriculum [4]; another is how to connect the lessons learned in the theoretical phase with their applications in practice [5]. Accordingly, the content of e-learning should be designed in accordance with the instructional needs of medical students to achieve a high-quality course. One strategy for such achievement is that the students determine their instructional needs themselves, identify the problems of e-learning systems, adopt necessary strategies for the success of their courses, and properly manage and guide their e-learning courses [6].

One of the novel and recent advancements in e-learning that is attracting the attention of higher education institutions is Massive Open Online Courses (MOOCs) [7], which are presented to an unlimited number of participants via the web. The University of Manibota first offered it in 2008 for connective information, expanding to 190 countries with about 160,000 learners [8]. A smooth supply of efficient instructions in the main key to improving the performance of learners; furthermore, the success of these courses is related to the interactive atmosphere in which students, instructors, and teaching assistants can participate, facilitated by discussion forums and instructional content such as videos, texts, and problem sets. The constructivist teaching modes in MOOCs aim at knowledge construction by the student rather than knowledge transfer from teacher to student. As the latter is the usual setting for medical education, using MOOC can offer an opportunity for educational innovation [9]. The materials are usually similar to university courses, but its advantages, including the fact that they are accessible to anyone and often do not have a specific starting and ending time, have resulted in increasing interest in this method of education [10]. Considering the recent coronavirus disease 2019 (COVID-19) pandemic, the significance of online education has increased. Therefore, it is necessary to investigate the needs of learning in each field of study.

Like other fields of study, medical education is also altering with the growth of digital platforms and educational methods, and MOOCs represent an excellent instructor-independent opportunity for the education of faculty members and students [11]. Such courses have been introduced as a possible solution to the challenges of medical education and have been welcomed by the world’s medical community [12]. The lack of geographical limitations, time limits, and subscription fees are important advantages of this educational method; the student only needs an internet-based device for learning [13, 14]. Accordingly, MOOCs are steadily increasing, and more free courses are being offered in health and medicine. Therefore, determining the role of MOOCs in medical education is vital [15]. Besides the advantages, MOOCs have some limitations, such as high dropout and low completion rates, content production expenses, and language barriers, though solutions have also been introduced to resolve these issues [13].

Despite the global attraction to MOOCs in medical education, the rate of participation is low in developing countries, and there is limited information about how medical students perceive MOOCs in these countries [16]. In our country, Iran, the high school graduate passes a national exam to enter university, and if accepted into a medical university, the students must pass a seven-year course (including internship) to graduate as a general practitioner. Most medical universities teach medicine to students via traditional methods [17], and MOOCs have been implemented in very few centers. Torbat Heydariye University of Medical Sciences launched e-learning through a learning management system (LMS) in 2014 and produced 34 MOOC items with the cooperation of Iran’s Virtual University of Medical Sciences (VUMS; established in 2017). ARMAN, the national MOOC platform, is the Persian abbreviation for new and massive national computerized education, which aims to produce MOOCs nationally and internationally. Since its establishment (2018–2019), several medical sciences universities have been cooperating with ARMAN and helped its development by holding workshops [18]. In line with these developments, more research is required to emphasize the importance of utilizing this novel technology, take effective steps toward its implementation [19], and identify challenges and related solutions [20], as presenting courses without a detailed analysis and prioritization of the needs would result in courses that fail to meet the actual needs of the target population, thereby generating expenses without causing any improvements in the knowledge, skills, and attitudes of learners. Hence, educational programs, especially in medical education, should be based on performance-related needs assessments to be motivating [21]. Therefore, the present study aimed to investigate the instructional needs of undergraduate medical students for presenting MOOCs to medical students of Shiraz University of Medical Sciences (SUMS), which can improve the quantity and quality of medical education.

## Methods

This needs-assessment study was conducted using the Delphi technique in three steps at SUMS during the 2020–2021 educational semester. In this technique, questionnaires and focus groups were used, group members’ comments were collected in special forms, and the resulting opinions were ranked based on priority.

The study population included basic and clinical faculty members, as well as medical students in their internships. Purpose-based and snowball sampling was used to select experts and instructors for this study. The inclusion criteria for the instructors were having a basic or clinical specialty, having passed their fellowship period within the previous five years, and familiarity with the key concepts in healthcare education. According to the inclusion criteria, 21 out of 403 faculty members in basic sciences and 68 out of 114 faculty members in clinical sciences were finally selected. Although the existing references consider an overall sample size of 30–60 for the Delphi technique [22], a larger number was taken considering the risk of sample loss. For the students, the statistical population consisted of all undergraduate SUMS medical students who started their internships (as an appropriate time to have acquired the necessary perspective on the instructional needs) in February or October 2014. The total number of students was 107, 67 of whom were selected through random sampling with a table of random numbers.

The Delphi technique is a straightforward means of determining the instructional needs of health and medical education systems by collecting and evaluating the opinions of individuals in a certain area [23], carried out in three steps:

**Round 1**: This step is the basic and most important step in the Delphi technique, in which we aimed to identify all instructional needs that can be met through MOOCs. The data collection tool in step one was a questionnaire containing demographic questions and one open-ended question designed for both instructors and students. The question asked: “What are the instructional needs that can be met through MOOCs for medical students?”. The researcher designed the question based on the study’s objectives after a thorough review of the relevant literature and confirmation by the study supervisor. After four experts in medical education and e-learning verified this questionnaire’s face validity and reliability, minor changes were made accordingly, and the final questionnaire was distributed among the faculty members and interns via Email, social networks, or instant messages. The respondents were asked to freely state their opinions about educational needs through brainstorming without prioritizing them. Considering the novelty of online education, a six-page PDF was also prepared that explained the online courses, MOOCs, and ARMAN (based on the available references) to the respondents.

The questionnaires were to be returned within seven days. The participants who had not returned their questionnaire were identified and followed through phone calls or social network messages, repeated (up to even nine times during one month) until an acceptable percentage of the questionnaires were returned. In some cases, in-person visits were made to complete paper-based questionnaires. Finally, 49 instructors and 47 students completed the questionnaires.

### Round 2

This stage of the Delphi technique was based on the responses to the questions in step one, aiming to identify the instructional needs. After analysis of the quantitative data collected in this step by MaxQDA software 2020, the viewpoints of some physicians and medical students were used to categorize these instructional needs and combine similar opinions based on the medical curriculum using text analysis. The results of this analysis were used to identify the educational needs related to online systems and MOOCs from the perspective of the instructors and students. This was changed to a structured questionnaire with a Likert scale, which was used as the tool for the second round. Two separate questionnaires were designed for the instructors and students. The instructors were asked to prioritize the instructional needs based on a 5-point Likert scale. Based on the instructional needs expressed by interns in step one, a separate questionnaire was designed for this group using the Likert scale.

The questionnaire designed for the instructors included 76 items, rated based on a Likert scale, while the questionnaire designed for the interns consisted of 56 items. Two open-ended questions were added: one for further suggestions, new educational topics, corrections, omissions, weaknesses, and strengths, and the other for asking about their agreement or disagreement. The respondents were also asked to state the reason for their prioritization. In step two, by providing feedback to the participants and expressing that the questionnaire is a continuance of the previous step, an effort was made to motivate the participants to continue their participation. A larger number of individuals completed the questionnaires in step two. The researcher-made questionnaire was rated based on a five-point Likert scale from 1 (*very low* priority) to 5 (*very high* priority). The face validity and content validity of the questionnaire were confirmed by several medical education experts; the reliability of the questionnaire was confirmed by delivering the survey questionnaire to ten members of the statistical population and calculating Cronbach’s alpha coefficient (0.964 for the instructors’ questionnaire and 0.931 for the students’ questionnaire).

The questionnaires were spread, like the previous round, and the respondents were asked to complete them within seven days; those who had not were followed for completion. Others were completed after in-person follow-ups (referring to the participant and asking them to complete a paper-based questionnaire). After one month, data from these questionnaires (49 instructors and 47 students) were collected and entered into SPSS Statistics software version 26, and the priorities were identified using descriptive and inferential statistics, as well as the nonparametric Friedman’s test.

### Round 3

The Delphi method classically involves four rounds, but researchers usually summarize them into three or two rounds to achieve their research objectives. The decision for the number of rounds depends on the time available to the researcher and the type of the initial question. Although validity increases with more rounds, the process often becomes tiresome after three rounds, and no new or useful results are achieved [24]. Therefore, in this stage, by utilizing a focus group methodology and inviting expert instructors, a final consensus was reached with regard to the opinions and priorities. A total of four expert faculty members at the Medical Education Development Center, two faculty members at VUMS, and one intern (graduate student in medical education) were invited to the focus group session. As the priorities of instructors and students differed, the collective wisdom of the experts in the focus group was used to achieve a consensus between the priorities. The criteria for achieving a consensus were determined first, and the common educational needs were analyzed using Friedman’s test. In the focus group meeting, first, the most common “expressed needs” of the Friedman table were selected according to the needs of instructors and interns. As the main audience of this education is students, we tried to set the students’ opinions as the first priority and the perspective of instructors and experts as the second priority. After examining the instructional needs prioritized by Friedman’s non-parametric test, the first ten priorities regarding the instructional needs presentable through MOOCs were identified. The validity of this questionnaire was reflected by Cronbach’s alpha values of 0.964 for the instructors and 0.931 for the students. The reliability of the items also showed sufficient precision of the questionnaire.

It should be noted that ethical requirements for data collection included informed consent, confidentiality, consent to participation, and the availability of researchers to answer any questions. Ethical approval for this study was obtained from the institute’s research ethics committee (IR.SUMS.REC.1398.969).

## Results

To analyze the data, descriptive and inferential statistics were applied. Among the faculty members and instructors, most respondents were males aged 35–45 years; most were married, and most had either a fellowship or postgraduate degree. Most interns were females aged 25–30 years, and most were single. In the first phase of the Delphi method, the basic instructional and clinical needs expressed by the faculty members were 266 titles; after analysis and classification, they were summarized into 36 instructional needs. The frequencies of the top 13 “expressed needs” are shown in Table 1. Furthermore, the instructional needs expressed by the interns included 311 titles; after analyzing and classifying them, they were reduced to 40. The top 13 instructional needs are listed in Table 1.

**Table 1:** Instructional needs expressed by instructors and interns (first phase of Delphi)

Row	Expressed needs (instructors)	Frequency	Expressed needs (students)	Frequency
1	Biochemistry	31	Physiology	29
2	Physiology	27	Anatomy	28
3	Anatomy	25	General lessons	26
4	Bacteriology	18	Biochemistry	25
5	Physical examination	17	Health	23
6	Cardiovascular diseases (theory)	17	Physical examination	22
7	Statistics and research methods	16	Case presentation & grand round	17
8	Virology	15	ECG interpretation	15
9	Specialized medical language	14	Interpretation of CT scans	13
10	Health	13	Cardiorespiratory resuscitation	12
11	Histology	12	Poisoning management	12
12	Endocrine diseases and metabolism (theory)	11	DKA, diabetes	11
13	Respiratory diseases (theory)	10	Convulsions	10

*Abbreviations* ECG; electrocardiography, CT; computed tomography, DKA; diabetic keto-acidosis

As shown in Table 1, the frequency of some topics of educational needs was very close to each other, including altered level of consciousness, internal medicine, respiratory infections in children, gastroenteritis, electrolyte disorders, trauma, and acute abdomen.

**Fig. 1:**
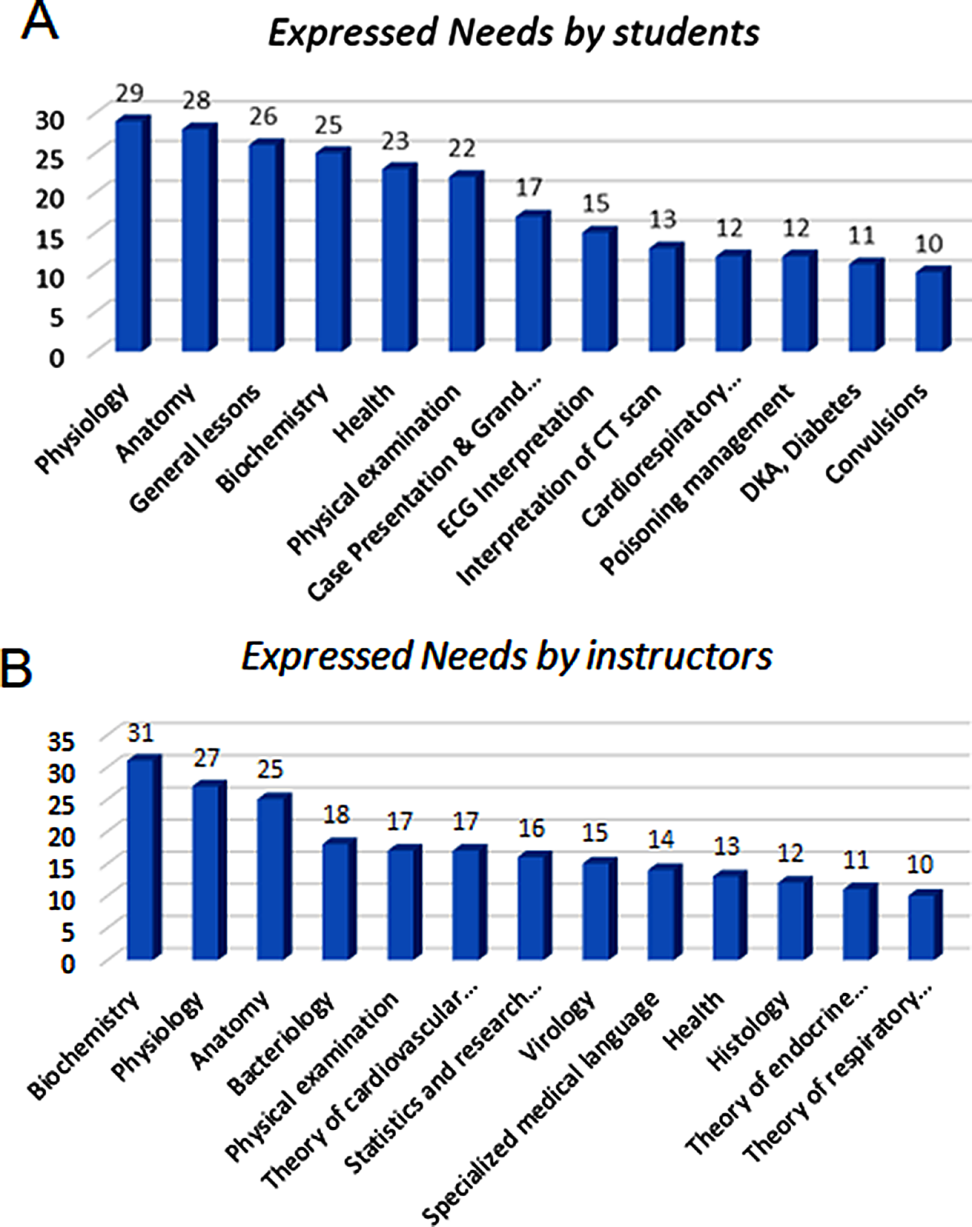
(**A**) Needs expressed by interns, (**B**) needs expressed by instructors

The needs expressed by interns and instructors, ordered based on frequency, are shown in Fig. 1A, B, respectively. These figures can help better understand the differences and similarities of the viewpoints of the interns and instructors.

After qualitative analysis of the questions, data analysis from the second phase of the Delphi questionnaire identified the first 20 needs of the interns and instructors regarding MOOCs. Table 2 shows the list of priorities with the highest scores, reported after applying Friedman’s test.

**Table 2:** Instructional needs expressed by instructors and interns after ranking with the highest score (the second phase of the Delphi method)

Expressed needs	Instructors (*n* = 49)		Expressed needs	Interns (*n* = 47)	
	Rank order	Mean rank (SD)		Rank order	Mean rank (SD)
Physical examination and history-taking	1	53.36 (0.79)	Common antibiotics and medications	1	37.44 (0.65)
Hypertension	2	51.93 (0.73)	Prescription	2	34.87 (0.89)
Familiarity with common drugs	3	50.73 (0.73)	ECG interpretation	3	33.69(1.04)
Professional ethics	4	48.52 (0.96)	Anatomy	4	32.67(1.15)
Principles and skills of patient care	5	48.30 (0.91)	Hypertension	5	32.52 (0.92)
Convulsions	6	48.28 (0.73)	Electrolyte disorders	6	32.28 (0.91)
Virtual learning skills	7	48.24 (0.88)	Acute bronchitis	7	32.16(1.03)
Virtual prescription training	8	47.26 (0.84)	Kidney diseases	8	32.12 (0.83)
Neurological examination	9	47.04 (0.81)	Pharmacology of opioids	9	32.09 (0.94)
Endocrine and metabolic diseases	10	46.92 (0.61)	Interpretation of radiographs and CT scans	10	38.82(1.01)
Asthma	11	46.51 (0.95)	Dermatology	11	31.37(1.10)
Case presentation & grand round	12	46.33(1.12)	Gastroenteritis	12	31.32 (0.75)
Pharmacology of opioids	13	45.88 (0.93)	Poisoning management	13	31.05 (0.93)
Respiratory diseases (theory)	14	44.43 (0.83)	Acute abdominal pain	14	30.97 (0.97)
Electrocardiography interpretation training	15	44.10 (0.99)	Diabetes and diabetic ketoacidosis	15	30.87 (0.82)
Headache	16	44.03 (0.84)	Internal diseases	16	30.85(0.72)
Anatomy	17	43.63(1.01)	Asthma	17	30.85(1.0)
Ischemic heart disease	18	43.20 (0.92)	Chronic obstructive pulmonary disease	18	30.84(1.05)
Physiology	19	43.14(1.03)	Physical examination	19	30.82(1.42)
Musculoskeletal diseases (theory)	20	42.46 (0.92)	Cardiovascular diseases (theory)	20	30.81(1.02)
Kendall’s W Test	0.195			0.072	
Chi-squared	715.584			186.186	
P-value	0.0001			0.0001	

*Abbreviations* ECG; electrocardiography, CT; computed tomography

Table 2 shows the distribution of the mean, standard deviation, and mean rank for the highest mean rank values, as expressed by the basic and clinical instructors and interns. The chi-squared value (715.584), significant at *P* < 0.01, indicates a substantial difference between the questionnaire questions in terms of importance. These questions were not of equal value and importance from the respondents’ point of view, and the observed distribution of ranks was not the output of a random factor.

The final step (focus group) identified “common medications (opioids and antibiotics), prescription medications, electrocardiography (ECG) interpretation, physiology, anatomy, electrolyte abnormalities, interpretation of radiographs and computed tomography (CT) scans, physical examination, heart disease and arteries (blood pressure and chest pain), and internal diseases (related to glands and metabolism, diabetes)” as the first ten priorities of instructional needs for MOOCs.

## Discussion

The present study identified the needs for medical education MOOCs by the Delphi method from the perspective of instructors and students. This study was performed in line with the main challenge of educational planning, tailoring the existing curriculum to the needs of diverse individuals and groups and improving the efficiency and effectiveness of the curriculum. The results of educational needs assessments can be used to enhance the quality of curricula and improve both the quantity and quality of medical education [25]. Considering the evolution of medical teaching methods, MOOCs have gained popularity in several fields, including medical education. However, as the system of medical education is different in Iran, MOOCs and other online methods are not popular in our country. Therefore, our groundbreaking study presents several interesting points for designing an appropriate MOOC for medical students in the Iranian educational system.

In this study, the perspectives of instructors and senior students (interns) were collected to identify the priorities. The results of the three-step Delphi method showed that the viewpoints of instructors and students had some similarities and some differences; the different educational needs expressed may be possibly due to different needs and attitudes. Many faculty members believe that MOOCs cannot replace a teacher, considering the lack of interaction with the instructor, while it is welcomed by most students, who hold that MOOCs can act as an interactive global community [26]. Therefore, it is important to collect the perspectives of both students and instructors. However, few studies have considered this in the needs assessment of MOOCs in medical education, and none have focused on the needs assessment of the educational curricula. Some researchers have focused on the quality of MOOC performance [11, 27], while others have examined the learners’ feedback from an anatomy MOOC [28]. This is while a needs assessment can help instructors prepare the health trainees to meet the needs of future patients better by including the prioritized needs in online curricula [29]. The few studies investigating the curricula have focused on providing MOOCs to health professionals in one specific area, like malnutrition [30] or substance abuse [31], rather than the general curriculum provided at a medical college. Others have focused on other aspects of medical education, like ethics, communication skills, and time management, rather than clinical courses [32].

The results of the present study showed that the similarity in the opinions of the instructors and interns included the prioritization of the interpretation of ECG, radiographs, and internal diseases among the first ten priorities of educational needs that can be met through MOOCs. As our literature review revealed no study with similar objectives to ours (need assessment of medical education MOOCs), the discussion is presented with studies that have evaluated the need assessment of medical education through other methods. In a study at Isfahan University of Medical Sciences, researchers evaluated the educational needs of third-year medical students and indicated the following courses as the major educational needs for the students: familiarity with common para-clinical examinations (ECG), major emergencies (altered level of consciousness, poisoning, fractures), resuscitation skills (such as cardiopulmonary resuscitation), familiarity with imaging interpretation (MRI, CT scan, X-ray), history-taking, physical examination, and medication prescriptions [33]. Despite differences in the educational methods, it can be claimed that these findings are quite consistent with the prior educational needs identified in the present study. In another study on third-year medical students, the results showed a clear desire for training in sonography, central line placement, paracentesis, and thoracentesis [34]. The difference between their results and that of the present study may be due to the different study populations, as they also evaluated residents’ perspectives.

Several studies evaluated the perspective of physicians rather than students. Consistent with our findings, the physicians of Ahvaz (a city in West Iran) prioritized internal and emergency medicine, imaging interpretation, and interpretation of para-clinical test results among the top ten priorities [21]. However, they outlined ear-nose-throat diseases, skin diseases, and gynecologic diseases as the top three priorities of educational needs [21], diverging from our results. This difference could be due to the varied preferences of physicians from students and instructors [35], as students’ viewpoints were considered the first priority in the present study. In North Khorasan, the top instructional priorities of general physicians included cardiovascular diseases, cardiopulmonary resuscitation, and diabetes [36], aligning with the present study’s findings. Another study evaluated the educational needs of general practitioners working in healthcare departments of the Ministry of Health and Medical Education using the Delphi technique. It concluded that diabetes, evaluation and accreditation of hospitals, communication skills for managers, principles of nutrition counseling, and neonatal resuscitation were among the top priorities, with diabetes and resuscitation also prominent in our study [37]. Family medicine workers in Iran also identified cardiovascular diseases and hypertension, diabetes prevention and management, and interpretation of ECGs among the top educational needs [38], which is consistent with the results of the present study. Other studies have also evaluated the education needs in specific fields of medicine, such as neurologic diseases [39], from the perspective of physicians or have assessed needs related to continuing medical education (post-graduate) [40, 41], falling outside the scope of our study.

Other interesting points of the present study were revealed by comparing different subgroups participating in the study; a comparison of the results of the first and the second quantitative rounds revealed that the educational needs stated by the students in the first qualitative round were slightly different from the top twenty educational needs. However, the top twenty educational needs stated in the second round were urgent needs. Interestingly, the students of different groups had almost the same educational needs; however, some of the educational needs stated by instructors from a specific clinical group could be different from the needs stated for other clinical groups, and each group mentioned the educational needs related to their own field of specification.

Another interesting point in the present study is that instructors believed that holding online courses or MOOCs cannot be effective for the clinical training of medical students. This opinion may result from the fact that MOOCs and e-learning are new in our country and not applied frequently; therefore, the different aspects of this type of education are not clear to the instructors, which might result in a negative attitude toward e-learning. Our findings are consistent with previous ones, stating that learning assessment in clinical settings in a MOOC format could be challenging for some medical educators, especially the older ones [11]. Younger instructors, however, considered MOOCs very effective along with clinical training.

The educational needs identified by faculty members and students in the present study were also related to knowledge and skills, like achieving professional competency in cardiopulmonary resuscitation and interpreting ECG and CT scan results. Nevertheless, it is not possible to achieve these high cognitive skills through e-learning and MOOC alone, and these modalities should be complemented by learning in clinical settings. This may imply that MOOCs cannot be suitable for all types of learning in medical education. Some have suggested that MOOCs can significantly impact learning and fill the gaps in formal education when used as a complementary, non-formal education [42]. Perhaps their combination would work best for medical students [43, 44]; however, more studies are required to determine the suitability of MOOCs and other online educational systems for medical students.

The present study had the main strength of evaluating a novel and highly applicable issue in medical education, which has not been evaluated in Iran’s educational system before. Other strengths included using the Delphi method for the survey on a large group, including both students and instructors, comparing their opinions. Despite these strengths, our study had some limitations. Firstly, the results of this study are basically based on the participants’ personal opinions, although participants with more experience were selected to get better results. Secondly, the opinions of one medical university in one city were evaluated, while differences may exist in other cities of Iran based on the medical needs of a city/province with dissimilar demographics and culture. Another limitation of the present study is related to the limited participation of instructors due to their unfamiliarity with online learning methods, including MOOCs; however, we provided them with a 6-page PDF to inform them about this educational method.

## Conclusion

In this study, we identified the educational needs of medical education through modern electronic educational technology, MOOC, and collected the viewpoints of medical students, clinical faculty members, and medical education experts. The findings of this study can be used in designing general medicine curriculum, revising instructional programs, adapting the curriculum to meet the needs of society, and continuing medical education to improve the quality and effectiveness of medical education; when other phases of instructional design, including monitoring and evaluation, are performed appropriately. Many reputable universities are now offering their courses in the form of MOOCs. Iranian authorities must provide the necessary grounds to create and utilize MOOCs in higher education in our country and provide the students with medical content designed based on their needs. It is recommended that further studies consider the viewpoints of participants who are familiar with online training methods, especially MOOC, and consider evaluating the effectiveness of the combination of offline (clinical) and online methods in medical education at several universities from different cities of the country.

## Data Availability

The datasets used and/or analyzed during the current study are available from the corresponding author upon reasonable request.

## Abbreviations

COVID-19: Coronavirus disease 2019
CT: Computed tomography
ECG: Electrocardiography
e-learning: Electronic learning
MOOCs: Massive open online courses
SUMS: Shiraz University of Medical Sciences
VUMS: Virtual University of Medical Sciences

## References

[1] Ghasemi M, Fardanesh H, Hatami J, Ahmady S (2018). Evaluation of the electronic learning system of medical education (case study of Shahid Beheshti Medical School). J Educ Strategies Med Sci.

[2] Naderifar M, Ghaljaei F, Jalalodini A, Rezaie N, Salar A. Challenges of e-learning in medical sciences: a review article. 9. 2016;23(102–11).

[3] McCutcheon K, Lohan M, Traynor M, Martin D (2015). A systematic review evaluating the impact of online or blended learning vs. face-to‐face learning of clinical skills in undergraduate nurse education. J Adv Nurs.

[4] Huwendiek S, Duncker C, Reichert F, De Leng BA, Dolmans D, van der Vleuten CP (2013). Learner preferences regarding integrating, sequencing and aligning virtual patients with other activities in the undergraduate medical curriculum: a focus group study. Med Teach.

[5] Ten Cate O, Durning S (2007). Peer teaching in medical education: twelve reasons to move from theory to practice. Med Teach.

[6] Lee T-H, Shen P-D, Tsai C-W (2010). Enhance low-achieving students’ learning involvement in Taiwan’s higher education: an approach via e-learning with problem-based learning and self-regulated learning. Teach High Educ.

[7] Joseph AM, Nath BA, editors. Integration of Massive Open Online Education (MOOC) System with in-Classroom Interaction and Assessment and Accreditation: An extensive report from a pilot study. Proceedings of the international conference on e-learning, e-business, enterprise information systems, and e-Government (EEE).; 2013: The Steering Committee of The World Congress in Computer Science, Computer &#8230.

[8] Al-Rahmi W, Aldraiweesh A, Yahaya N, Kamin YB, Zeki AM (2019). Massive open online courses (MOOCs): data on higher education. Data Brief.

[9] Hendriks RA, de Jong PG, Admiraal WF, Reinders ME (2019). Teaching modes and social-epistemological dimensions in medical massive open online courses: lessons for integration in campus education. Med Teach.

[10] Hew KF, Cheung WS (2014). Students’ and instructors’ use of massive open online courses (MOOCs): motivations and challenges. Educational Res Rev.

[11] Olivares Olivares SL, Hernández RIE, Corolla MLT, Alvarez JPN, Sánchez-Mendiola M (2021). MOOC learning assessment in clinical settings: analysis from quality dimensions. Med Sci Educ.

[12] Harder B. Are MOOCs the future of medical education? Bmj. 2013;346. 10.1136/bmj.f2666 23624666

[13] Subhi Y, Andresen K, Bojsen SR, Nilsson PM, Konge L (2014). Massive open online courses are relevant for postgraduate medical training. Dan Med J.

[14] Blum ER, Stenfors T, Palmgren PJ (2020). Benefits of massive Open Online Course participation: deductive thematic analysis. J Med Internet Res.

[15] Liyanagunawardena TR, Williams SA (2014). Massive open online courses on health and medicine. J Med Internet Res.

[16] Aboshady OA, Radwan AE, Eltaweel AR, Azzam A, Aboelnaga AA, Hashem HA (2015). Perception and use of massive open online courses among medical students in a developing country: multicentre cross-sectional study. BMJ open.

[17] Firoozjahantighi A, jokar F, Haghani F, Ahmadnia S (2022). Generational characteristics of general medicine students in Iran. J Educ Health Promotion.

[18] Keshavarz M, Ghoneim A (2021). Preparing educators to teach in a digital age. Int Rev Res Open Distrib Learn.

[19] Ghazi Mirsaeed SJ, Ommati E (2017). Comparative survey of MOOC presented on Maktab khaneh website based on quadruple indexes focusing on the field of medicine. Payavard Salamat.

[20] Zeinabadi HR, Musavi AT (2017). Investigate the status of education based on mooc in Iran’s higher education; challenges and solutions. Quartely J Innov Enterpreneurship.

[21] Baghaei S, Rajaei E, Shokouhi A, Hasanian A, Sahraee M, Ehterami A (2019). [Investigation of priorities and NeedAnalyses of Instructional Programs for GP subjected to continuous retraining courses in Ahvaz]. Educational Dev Jundishapur.

[22] Adhami A, Haghdoost AA, Darvishmoqadam S, Nouhi E (2000). Determining valid criteria for evaluating clinical and theoretical teaching of the faculty of Kerman University of Medical Sciences. Iran J Med Educ.

[23] Wasfy NF, Abouzeid E, Nasser AA, Ahmed SA, Youssry I, Hegazy NN (2021). A guide for evaluation of online learning in medical education: a qualitative reflective analysis. BMC Med Educ.

[24] Ahmadi M, Zakerian SA, Salmanzadeh H, Mortezapour A (2016). Identification of the ergonomic interventions goals from the viewpoint of ergonomics experts of Iran using fuzzy Delphi Method. Int J Occup Hygiene.

[25] Price DW, Wagner DP, Krane NK, Rougas SC, Lowitt NR, Offodile RS (2015). What are the implications of implementation science for medical education?. Med Educ Online.

[26] Sitzman KL, Jensen A, Chan S (2016). Creating a global community of learners in nursing and beyond: Caring Science, mindful practice MOOC. Nurs Educ Perspect.

[27] Hartzell JD, Yu CE, Cohee BM, Nelson MR, Wilson RL (2017). Moving beyond accidental leadership: a graduate medical education leadership curriculum needs assessment. Mil Med.

[28] Swinnerton BJ, Morris NP, Hotchkiss S, Pickering JD (2017). The integration of an anatomy massive open online course (MOOC) into a medical anatomy curriculum. Anat Sci Educ.

[29] Chen BY, Kern DE, Kearns RM, Thomas PA, Hughes MT, Tackett S (2019). From modules to MOOCs: application of the six-step approach to online curriculum development for medical education. Acad Med.

[30] Eglseer D. Development and evaluation of a massive Open Online Course (MOOC) for healthcare professionals on malnutrition in older adults. Nurse Educ Today. 2023:105741. 10.1016/j.nedt.2023.105741 36746061

[31] Monteiro EP, Gomide HP, Remor E (2020). Massive open online course for Brazilian healthcare providers working with substance use disorders: curriculum design. BMC Med Educ.

[32] Javaeed A. General needs assessment of the undergraduate medical students to integrate courses on medical ethics, time management and communication skills into the bachelor of medicine, bachelor of surgery curriculum of Pakistani medical colleges. Cureus. 2019;11(4). 10.7759/cureus.4433 PMC6559698 31245220

[33] Dadgostarnia M, Vafamehr V (2014). [Assessment of training needs of student’s clinical skills prior to entering the clinical course and impact of preliminary courses of training clinical skills on students]. Iran J Med Educ.

[34] Kessler C, Bhandarkar S (2010). Ultrasound training for medical students and internal medicine residents—a needs assessment. J Clin Ultrasound.

[35] Weitz G, Twesten C, Hoppmann J, Lau M, Bonnemeier H, Lehnert H. Differences between students and physicians in their entitlement towards procedural skills education–a needs assessment of skills training in internal medicine. GMS Z für Medizinische Ausbildung. 2012;29(1). 10.3205/zma000777 PMC3296102 22403592

[36] Rajabzadeh R, Jabari N, Saadati H, Alavinia SM, Jalilvand M, Hosseini ME (2017). [Training Needs Assessment for General Practitioners Engaged in North Khorasan University of Medical Science]. Educational Dev Jundishapur.

[37] Modiri F-K, Alavinia SM, Labaf-ghasemi R, Shams M (2012). [Educational needs Assessment of General Physicians Working. Teb-va-Tazkiye.

[38] Kabir MJ, Ashrafian Amiri H, Rabiee SM, Momtahen R, Zafarmand R, Nasrollahpour Shirvani SD (2018). [Educational needs of family physicians and health care providers working in the family physician program of Iran]. Med Educ J.

[39] Vakilian A, Iranmanesh F, Shafa MA, Moghadam-Ahmadi A, Maleki-Rad F (2015). [Educational needs Assessment for General practitioners in the field of neurological diseases in the Regulatory Zone of Rafsanjan University of Medical Sciences Iran]. Strides Dev Med Educ.

[40] Ratnapalan S, Hilliard RI (2002). Needs assessment in postgraduate medical education: a review. Med Educ Online.

[41] Norman GR, Shannon SI, Marrin ML (2004). The need for needs assessment in continuing medical education. BMJ.

[42] Cusumano MA (2014). MOOCs revisited, with some policy suggestions. Commun ACM.

[43] Doherty I, Sharma N, Harbutt D (2015). Contemporary and future eLearning trends in medical education. Med Teach.

[44] Mehta NB, Hull AL, Young JB, Stoller JK (2013). Just imagine: new paradigms for medical education. Acad Med.

